# A Fitness App for Monitoring Walking Behavior and Perception (Runkeeper): Mixed Methods Pilot Study

**DOI:** 10.2196/22571

**Published:** 2021-03-01

**Authors:** Justin B Hollander, Sara C Folta, Erin Michelle Graves, Jennifer D Allen, Minyu Situ

**Affiliations:** 1 Department of Urban and Environmental Policy and Planning School of Arts and Sciences Tufts University Medford, MA United States; 2 Friedman School of Nutrition Science and Policy Tufts University Medford, MA United States; 3 Federal Reserve Bank of Boston Boston, MA United States; 4 Department of Community Health Tufts University Medford, MA United States; 5 Graduate Program of Community and Regional Planning School of Architecture University of Texas Austin Austin, TX United States

**Keywords:** physical activity, smartphone, mobile app, sense of belongingness, community cohesion

## Abstract

**Background:**

Physical activity has a strong positive impact on both physical and mental health, and public health interventions often encourage walking as a means to promote physical activity. Social connectivity, such as that among spouses, families, friends, and colleagues, highly influences physical activity. Although technology-based interventions have some influence on human behavior, they have not been fully implemented and evaluated for their influence on walking through social connectivity.

**Objective:**

We aimed to pilot-test the organization of neighborhood walking clubs and use of a mobile app (Runkeeper) to encourage social connectedness and neighborhood cohesion, as well as to increase physical activity.

**Methods:**

We used a convenience sampling method to recruit 46 adults from an urban location in Greater Boston, Massachusetts. We assigned participants to teams based on their geographic location and neighborhood and required them to use the app (Runkeeper). Participants completed 2 self-administered web-based surveys before and after the intervention period. The surveys included standard measures to evaluate physical activity, social connectedness, perceived social support, and neighborhood cohesion (Buckner Neighborhood Cohesion Scale) before and after the intervention. Following the intervention, we randomly selected 14 participants to participate in postintervention, in-depth phone interviews to gain an understanding of their experiences.

**Results:**

This study was approved by the institutional review board in June 2018 and funded in January 2018. Recruitment started in May 2019 and lasted for 2 months. Data were collected from July 2019 to January 2020. In this study, Runkeeper was of limited feasibility as an app for measuring physical activity or promoting social connectedness. Data from the app recorded sparse and uneven walking behaviors among the participants. Qualitative interviews revealed that users experienced difficulties in using the settings and features of the app. In the questionnaire, there was no change between pre-post assessments in walking minutes (*b*=−.79; 95% CI −4.0 to 2.4; *P*=.63) or miles (*b*=−.07; 95% CI −0.15 to 0.01; *P*=.09). We observed a pre-post increase in social connectedness and a decrease in neighborhood cohesion. Both quantitative and qualitative results indicated that the psychosocial aspects of walking motivated the participants and helped them relieve stress. Interview results showed that participants felt a greater virtual connection in their assigned groups and enhanced connections with friends and family members.

**Conclusions:**

Our study found that Runkeeper created a virtual connection among walking group members and its data sharing and ranking motivated walking. Participants felt that walking improved their mental health, helped to relieve stress, and made them feel more connected with friends or family members. In future studies, it will be important to use an app that integrates with a wearable physical activity device. There is also a need to develop and test intervention components that might be more effective in fostering neighborhood cohesion.

## Introduction

### Physical Activity

Physical activity (PA) has been found to be significantly associated with not only physical health and chronic disease risk reduction [1] but also with psychological health and well-being [2,3]. The World Health Organization calls for interventions to promote PA as a means of lowering the global burden of chronic disease, with group walking thought to potentially increase the motivation to engage in PA [4,5]. Despite the known health benefits of PA, just over half of the adults in United States (51%) meet the 2008 federal guidelines for PA [6]. Many practitioners have focused on walking as a form of PA that is inexpensive, accessible, and well accepted among adults [7-9]. Walking is the most common form of PA in the United States [4]. It has a low risk of injury [10], making it safe for all age groups and feasible even in the presence of other health concerns or logistical barriers to exercise. Interventions to increase moderately intense walking among Americans could help meet the PA guidelines [11].

In general, walking has multiple positive effects on health and well-being. It improves physical health by improving blood pressure control [12-14], weight loss [12,15], and prevention of obesity and cardiovascular diseases [16-18]. Research has shown that walking can affect other dimensions of well-being, such as reducing depression [19], lowering physiological stress [20], and stabilizing cognitive functioning for those at risk of dementia [21]. Sedentary people can benefit from modest increases in walking, especially for otherwise healthy, middle-aged or older adults.

### Group Walking

There is some evidence that walking in groups influences the motivation to walk. Ball et al [22] found that the likelihood of an individual walking for physical exercise increases when they are doing so with another person. People also prefer and enjoy walking with others more than walking alone [8]. The benefits of PA are realized through group walking. Meta-analyses of walking group interventions showed statistically significant improvements in blood pressure, resting heart rate, and body mass index, among other measures [12,23]. Most of the studies report on the physical benefits of walking in groups [23], with some research measuring emotional well-being. These generally show a reduction in depression scores [12,24], although one recent study found that individual walking is more effective at reducing depression [25]. Marselle et al [11] found that walking in groups contributed more to personal emotional and mental well-being than walking individually.

Walking clubs can potentially improve social connectivity [26]. Chen and Pu [26] reported that walking in teams promotes social connections with friends. Several recent studies also found positive effects for participating in walking clubs, including among specific populations including postpartum women [27], adults with diabetes, and older adults [28-30]. A number of researchers have reported that the social dimensions of a walking group contribute to people’s interest in the initialization of walking, maintaining participation, and increasing walking behavior [31,32]. Others raise concern that walking clubs may disproportionately serve traditionally advantaged subpopulations, although walking clubs in the United States may be relatively more inclusive than other countries [33].

Although walking programs have the potential to improve *social* well-being, defined as a sense of belonging and interdependence with others [34], the impact of group walking on social well-being is understudied relative to the larger literature on physical well-being. Qualitative research suggests that group walks have a positive effect on social well-being [35,36]. In their meta-analysis, Meads and Exley [23] report that *walking in groups tended to increase quality of life measures and may increase social connectedness, but the evidence for this was uncertain* [23]. Thus, although the literature supports the conclusion that walking clubs offer a number of individual health and psychological benefits, the social benefits are less well established.

Recent technological interventions have been shown to increase social connectivity and PA. However, few studies have explored the use of technology to facilitate neighborhood walking clubs as a PA intervention. Walsh et al [37] conducted a study to analyze the effect of mobile phone app intervention on increasing daily walking steps among youth. Their study pointed out that PA could be enhanced through specific settings in mobile phone apps, such as self-monitoring of walking steps and setting of personal walking goals. Chen and Pu [26] used a mobile app called *Healthy Together*, which enabled users to participate in physical activities together by sending each other messages and earning badges. Their research goal is to compare different social incentives in mobile fitness apps and to provide new angles of design implications for mobile fitness apps. They found that building users’ performance with their team members promotes not only individuals’ PA levels but also social connections with friends. They recommended that an app design for physical activities should consider adopting social interaction as the key motive for user involvement [26]. Some recent innovative electronics such as *FitBit* and *Jawbone* are wearable PA tracking technology, which enable users to connect their data of physical activities to their phone or app automatically [38]. Comparison to others could be perceived as motivating if people looked to those with greater PA success as positive role models, which is an upward comparison [38]. Women who had a strong interest in upward comparisons also presented significant increases in PA [39].

However, most of the above studies mainly focused on the benefit and motivation of walking at the personal level, instead of focusing on whether the walking clubs impact neighborhood social cohesion.

This study aims to examine the potential impact of walking clubs on neighborhood social cohesion. The goal of this study is to gather preliminary information on technologically enhanced walking clubs to encourage social connectedness and neighborhood cohesion and to increase PA to help guide future studies that will advance public health not only at the personal level but also at the community level.

## Methods

In this mixed methods pilot study, we used a pre-post evaluation design to assess the potential impact of the intervention Runkeeper on walking distance and duration, belongingness, neighborhood cohesion, motivation for PA, and self-reported PA. Qualitative methods were used to contextualize quantitative results. This study was approved by the institutional review board in June 2018 and was funded in January 2018. Recruitment started in May 2019 and lasted for 2 months. Data collection started in July 2019 and ended in January 2020.

### Sample and Setting

The inclusion criteria were living in an urban location in Greater Boston, Massachusetts, aged 18 years or older and ability to speak and read English. We used geographic information systems and census track-level demographic and socioeconomic data to identify neighborhoods for the walking clubs, with the aim of reaching a diverse set of participants across income, race, ethnicity, and age. The final locations were determined based on the number of potentially eligible participants and their proximity to Tufts University.

The procedure involves 4 main steps. First, participants were recruited and administered a mandatory baseline survey (May-June 2019); the second step was to monitor participants’ walking data coming from the app for 3 months (July-October 2019); the third step was conducting a mandatory exit survey to gain participant feedback (October 2019); and the last step was interviewing participants in terms of the data reflection (January 2020). The baseline survey and exit survey took participants 30 min to complete, and the questions in the baseline survey were identical to all questions in the exit survey, and they were both 9-page surveys and each page had 4 questionnaire items.

Participants were recruited through Facebook advertisements and door-to-door invitations in the neighborhoods of interest. Recruitment took 1.5 months, which involved recruiting participants and administering a baseline survey (May 21 to June 30, 2019). When potential participants responded to our Facebook advertisement, we would confirm their home addresses first to see whether they were in our desired location before sending them informed consent agreement. If they agreed in person (during door-to-door recruitment), they signed a hard copy of the informed consent agreement and provided an email address for further contact. No personal information was collected. Upon study entry, each participant was given a unique study identification number, which was only for data organization, management, and analysis. This identification number was associated with all data records from both surveys and apps. Participants’ names or other identifying information was kept separate from this identification number. The survey was hosted and all online data were stored on a secure server, and the hard copy of the consent form was stored in a locked office.

After recruitment, our researchers were in charge of inviting participants to take both baseline and exit surveys by sending emails. We used an open survey approach where the survey links sent to participants were the same, and the technical functionality of the electronic questionnaire was tested before fielding the survey via Qualtrics. Participants were able to change their answers through a back button, which displays a summary of the responses. The survey never displayed a second time once the participants had completed it. In addition, only completed surveys were analyzed, and the corresponding participants were allowed to continue accepting the walking club intervention.

During the baseline survey, all eligible participants received the walking club intervention. We purposefully divided them into 3 groups, of which the geographic radius of each group was within a walkable distance (10-min walking time). Each group had an average of 15 participants. Each group was asked for a volunteer to serve as a captain. The captain was responsible for serving as a liaison with the Tufts research team and organizing several walks per month for the group. Recent research has proved that the frequency of communication across teams is significantly associated with maintaining walking steps [40]. A participant volunteered to be captain in two of the groups, and the study coordinator served as the captain in the third group. In addition, participants received a US $100 gift card for completing both pre- and postsurveys and for 3-month monitoring of walking, as tracked by Runkeeper. Those who participated in phone interviews following the intervention were provided with an additional US $50 gift card.

Walking clubs were facilitated using Runkeeper, a free mobile fitness app that allows users to keep track of distance traveled, calories burned, and time spent engaging in physical activities. The app separates statistics by week, month, and year so that people are able to track their short- and long-term progression. In addition, Runkeeper has social features that set it apart from other fitness trackers. It is possible to create groups within the app in which the members can interact. Users can scroll through a feed and leaderboard that details their group mates’ PA, and they can like, post, and comment. A feature of the app is that it is necessary to manually start and stop exercise occasions for the app to log them. Participants were required to log walks on Runkeeper at least twice per month (6 times in total) to be considered adherent to the intervention.

### Quantitative Data Collection

Runkeeper uses GPS technology to record walking distance in miles (not steps). Participants manually started and stopped the app for each PA occasion by selecting a start function on the app to track walking time data. The research team had access to app data on the aggregate minutes and miles walked per participant for each of the 12 intervention weeks. Data were analyzed using a mixed effects regression model with participant ID as the random effect. The survey data were analyzed using paired sample one-tailed *t* tests. Stata version 15 was used for all analyses. We also had data from the app on the number of walks that occurred each week. These data were used to help classify participants as adherent or not, defined as at least two walks per month. Sensitivity analyses were conducted using data from adherent participants.

Before and after the 3-month intervention period, all participants received a web-based mandatory survey (Qualtrics). Self-reported PA was measured using the International Physical Activity Questionnaire (IPAQ), long form, which provides an estimate of walking, moderate-intensity, and vigorous-intensity activity within each of the following domains: work, transportation, domestic chores and gardening (yard), and leisure time [41]. Metabolic equivalent (MET) minutes per week were compiled according to the IPAQ’s Guidelines for Data Processing and Analysis [42].

The pre-post survey included questions about motivation for PA using the Physical Activity and Leisure Motivation Scale [43]. Its 8 subscales are (1) competition or ego, (2) appearance, (3) other’s expectations, (4) affiliation, (5) physical condition, (6) psychological condition, (7) mastery, and (8) enjoyment. Each subscale has 4 items, measured on a 5-point Likert scale ranging from 1 (strongly disagree) to 5 (strongly agree). Higher scores reflected higher motivation levels.

Social factors were also included in the pre-post survey. Social connectedness was measured using the General Belongingness Scale [44]. This scale has 2 domains, each with 6 items: acceptance or inclusion and rejection or exclusion. The rejection or exclusion subscale is reverse-coded so that higher scores reflect greater levels of perceived acceptance or social connectedness. Neighborhood cohesion was measured using the Buckner Neighborhood Cohesion Scale [45]. This validated scale has 18 items that measure 3 constructs: attraction to neighborhood, neighboring, and psychological sense of community [46,47]. *Attraction to neighborhood* measures resident’s degree of willingness to remain in the neighborhood, *neighboring* is the perceived degree of interaction between residents in the neighborhood, and *psychological sense of community* generally refers to the subjective sense of community that residents felt within the neighborhood [45].

We ran tests of significance to ensure internal validity of the data collected from both surveys and apps. First, we ran descriptive statistics and all variables of interest. Then, we ran the inferential statistics (regression) to explore the cause of the relationship between the independent and dependent variables.

### Qualitative Data Collection

We used postintervention qualitative interviews to help assess engagement with the intervention, usability of the app, and contextualize the quantitative results. On the basis of the 3-months monitoring and final data collected from the Runkeeper and surveys, we randomly chose 14 participants to conduct a phone call interview. The interviewees included 2 captains, 2 active (met adherence criteria) and 2 inactive (did not meet adherence criteria) participants in each group. The interviews were conducted in January 2020 and averaged 25 min in length. All participants were asked to conduct a phone interview with our research assistants through the FreeConferenceCall app [48]. The audio content of each interview was recorded with the permission of the interviewees.

Our recorded audio files of the interviews were transcribed verbatim using GoTranscript [49]. The data were analyzed using qualitative content analysis [50,51]. The initial codebook was mainly based on the questioning route and was refined based on team discussion. The final themes were built based on the patterns and frequencies found in the data, and all final codes were established at 90% agreement. NVivo (version 12, QSR International) was used to assist in the process of coding and analysis.

## Results

### Final Sampling

In total, we recruited 46 participants: 34 from the Facebook advertisement, 5 from door-to-door recruitment, and 7 from friends’ recommendations. A total of 7 were identified as males and 39 as females (Multimedia Appendix 1). The participation rate of the baseline survey was 100% (43/43), and the completion rate of the exit survey was 100% (43/43).

### PA

On the basis of the data collection from the app, the results indicated no increase in either minutes (*b*=−.79; 95% CI −4.0 to 2.4; *P*=.63) or miles (*b*=−.07; 95% CI −0.15 to 0.01; *P*=.09) walked over the intervention weeks. According to the IPAQ, there was a significant decrease in moderate MET minutes per week from pre- to postintervention; no other intensity categories were significant (Multimedia Appendix 2). In the sensitivity analysis, pre-post total and walking MET minutes per week were positive but remained nonsignificant. The pre-post change in psychological condition as a form of motivation (engaging in PA to relax and cope with stress) was positive and significant (Multimedia Appendix 2). There were no significant changes in any of the other types of motivation. Changes in physical condition and affiliation (doing it with others) motivations changed direction in the sensitivity analysis and remained nonsignificant.

### Qualitative Analysis

Themes from the interviews provide further insight into walking and motivation (Multimedia Appendix 3). Although not reflected in the survey data, one theme suggests that participants were motivated by the competitive aspect of the walk ranking on the app. Participants also reported that being organized in groups to walk enhanced feelings of accountability, competitiveness, and peer pressure.

I felt a little positive peer pressure.

I feel like when I had to be accounted for it, I did more, but when I wasn't being accounted for it, I did less.

A theme from the interviews supports and provides context for the change in psychological condition as a form of motivation. Many considered walking to be the best way of releasing stress, especially after their daily work.

Anytime I've had maybe an argument with someone or just had a stressful day, like going for a walk does help.

In addition, many participants described walking as *meditative* and described inner peace as the most meaningful benefit of walking.

It was nice because it's very peaceful. Walking, to me, is almost meditative. You get to be in the moment. I guess you can be mindful.

Themes from the interviews further suggest that participants were avid walkers but not avid app users. Many said that they walked more frequently than suggested by the app and often forgot to manually start it.

I probably used the app 25% of the time while walking.

This was a particularly strong theme among participants who did not meet the adherence criteria.

I would forget about [using] it, that would be the honest answer...Then I would realize, what I'm sending you guys is probably just a small percentage of what I actually do because the app didn’t catch my walk accurately.

Although many participants appreciated the tracking features of the app, most were frustrated by the manual operations of it.

I would forget to turn it on or turn it on midway or turn it off for one part and then I would stop walking and then forget to turn it back on.

Some said they preferred wearable PA monitors that did not require this. Themes related to the functions and operation of the app were similar between the adherent and nonadherent groups. However, nonadherent participants described more barriers to PA, such as work and family issues.

I would say that I tried to walk every week, or at least every two weeks...I am just too busy sometimes with the two kids to fit in that, to fit in a walk. I guess it would be more of a time thing more than a not wanting to.

General belongingness increased significantly from pre- to postintervention, whereas neighborhood cohesion decreased (Multimedia Appendix 2). The total scores and all subscores were significant for both measures. There were no changes in direction or significance based on the sensitivity analysis.

In the interviews, a theme from both adherent and nonadherent participants was that they enjoyed walking by themselves. Nonadherent participants did not mention walking with others. Adherent participants said that they preferred to walk with friends or family members if they needed companionship.

I would walk with my mom.

I walked with that one friend every time.

When they walked with others, it was described as an opportunity to enhance these established relationships.

It was nice because our girls could catch up on what’s going on with our lives...I think it made us more connected because usually when we get together, we go out once a week, but this time, we were getting together two, three times a week...

Regarding neighborhood cohesion, participants mostly expressed that walking enhanced their appreciation for the physical and natural features of their neighborhood.

I like looking at people's houses, backyards or front yards and things like that. So, I do feel a greater sense of belonging to my neighborhood or connection to my neighborhood.

Some participants also felt more virtually connected with their neighbors because of the chat platform on the app.

I feel a little bit more connected to the neighborhood because the people that were in my group that we spoke during tech-wise lived around you.

These participants also revealed a lack of belongingness to their physically existing community.

I also feel like I might be in a little bit of a different mindset since I’m a college student and don’t really view [city] as my home, home. Whereas some of the people in the study, this is their full-time residence and they know their neighbors and things like that.

## Discussion

In this study, we deepened the insight into the social and health benefits derived from participating in a neighborhood walking club facilitated by a technical intervention.

According to the questionnaire, there was no change between the pre-post assessments in walking minutes or miles. However, both quantitative and qualitative results indicated that the psychosocial aspects of walking motivated them and helped relieve stress. The interview results showed that they considered the most meaningful benefit of walking outside to be that it allowed them to relieve daily stress and find some form of inner peace. Their feedback is consistent with recent research that outdoor walking is a way of restoring mood and releasing stress [52,53].

### Walking Patterns and Technology

Neither app data nor self-report (IPAQ) indicated an increase in PA. However, based on interviews, the need to manually start and stop the app resulted in inaccurate logging of activity time. This finding is aligned with suggestions raised in other studies that an app is best designed to integrate with wearable PA tracking devices [26,38]. In addition, interviewees described themselves as avid walkers, suggesting a ceiling effect that would make it difficult for the intervention to further increase walking behavior. Preintervention MET minutes of activity indicated a highly active cohort. Even *nonadherence* likely refers to nonuse of the app rather than inactivity, based on the qualitative results.

### Social Connectedness and Technology

Research suggests that with respect to external interventions that try to modify human habits, people are generally interested in changing their behavior in a particular environment only if they find that their energy and time are not wasted and that there are more benefits or motivations generated. In our sensitivity analysis, the motivation of the competition and ego did not change, but our qualitative results showed how their motivation for competition played a role in their participation in walking. Interviewees’ motivation to increase walking was stimulated by the sharing of ranking data in their corresponding group on the app, which generated feelings of competitiveness. This is consistent with the findings of Chen and Pu [26] that linking users’ performance with their teammates promotes PA. The feature of ranking in the app provides a solution in dealing with *the absence of effective tools in motivating members in walking clubs* [54].

Interviewees’ reflections on walking data sharing and ranking are consistent with the literature that sharing PA data and ranking among groups in apps stimulates social comparison [55-57]. In addition, according to the findings of Arigo et al [39], women’s strong interest in upward comparison motivates their physical activities. Our interviewees were all female, and some of them who had a feeling of competition were from our active app users. Comparing individual ranking data in a group increases individuals’ likelihood of PA, which provides another possibility of PA motivation: that social support comes from not only those they are familiar with [58] but also from social cohesion through community connections [59].

In our quantitative analysis, neighborhood cohesion significantly decreased from the beginning of the study to the end, whereas general belongingness increased. On the basis of the qualitative analysis, our interviewees did not have a strong feeling of connectedness to their physical communities, although they felt a greater virtual connection in their assigned groups. The qualitative results provide an explanation for these results. Although our entire walking club was built at a geographic community around Tufts University, group members relied on the virtual connection generated from the chat feature on Runkeeper rather than meeting in person for walks. This is consistent with the argument raised by Gusfield [60] that community can also be defined by *human relationship* rather than geographic boundaries. The feeling of virtual connection in our study should be noted as a new *sense of community*, which is related to the findings from McMillan and Chavis [61] that a *sense of community* would be shaped by feelings of membership and shared emotional connection. In addition, the walking incentive generated by the ranking competition is consistent with the argument raised by Wood et al [62] that walking for purpose was associated with a sense of community. The walking clubs built in our study and the mechanism of ranking in the app may create a feeling of membership and shared competitive connection for our participants, which might also result in the *sense of online community* coming up. However, as members know that their online community was also all residents of the same geographic communities, it is not possible to know if the sense of online community would have developed without the underlying knowledge of shared geography.

Interviewees indicated that they felt a physical connection with their neighborhood, which would not be particularly well captured by the Neighborhood Cohesion Scale. None of our interviewees indicated feeling physically closer to their fellow club members who were strangers to them before the study. Rather, they did feel close ties with friends or family members in real life after this study, even though their friends or family members did not participate. In our sensitivity analysis, the motivation of the affiliation (ie, walking with others) motivations changed direction. This reflects that participants tend to stay with their familiar connections, which validates the research conducted by Chen and Pu [26], who recruited people and their friends together to increase participants’ motivation for involvement. Chen and Pu [26] pointed out that technical interventions for physical activities should consider adopting social interaction. On the basis of our interviewees’ experience, social interaction in PA should be built upon established relationships rather than the interactions of strangers, a point that has been overlooked in previous literature.

### Limitations

Given that the number of participants was limited in this pilot study, our findings cannot be generalized, and selection bias cannot be ruled out. Although our chosen mobile app has fully accessible open data, it had complicated settings and no auto-tracking, which led to inaccurate data. In addition, the intervention period lasted for 3 months during summer, and 3 months may not be long enough to observe a measurable impact on behavior. However, the fact that the intervention took place in summer may overestimate walking behavior, as people are more active in the summer. The long-term effects of community-level cohesion and participants’ various walking patterns and motivations throughout the year could not be tracked in this study.

### Conclusions

This pilot study aims to gather preliminary information on technology-based interventions for walking to promote social connectedness and neighborhood cohesion and to increase PA.

According to participants’ feedback, walking benefits were not exclusive to physical health, as mental health and inner peace were addressed as well. Given that not only our study but also other scholars have observed that walking has an impact on mental health improvement, the effective promotion of walking clubs should be related to the latest topics in mental health and stress release. The number of participants recruited from Facebook was much larger than that from on-site recruitment, which indicates that traditional recruitment was not as effective as the internet-based method. The mechanism of recruitment conducted by Chen and Pu [26], asking participants to invite at least one person to join them, had some success in this study.

By adopting the intervention of a mobile app, the *sense of online community* appeared in our findings; it appeared that place-based connections that might enhance neighborhood cohesion were not formed. The feeling of competitiveness derived from online data sharing was found in our study as a new incentive for walking. However, it was not the direct motivation for social connections among group members. The mobile app created a virtual connection among walking group members, and its data sharing and ranking generated new motivations for individual walking. However, it was difficult to build social connections effectively in walking clubs. On the basis of our qualitative analysis, the ties between participants and their friends were enhanced because they walked together, but they did not develop connections with other participants who were strangers to them before the study.

Technology-based interventions should be tailored to the majority’s needs. Participants in our study expressed their need to use wearable auto-tracking fitness app or electronic and push notifications. The mobile app should be provided with communication timeliness and user convenience, which would increase the chance of changing human behavior of walking and social contact regarding technology-based interventions. Ogilvie et al [63] concluded that people would be able to walk more if interventions were targeted at their needs or motivations.

In summary, the technology-based intervention for walking effectively boosts individual-level well-being by creating virtual connections and feelings of competitiveness. Most participants discovered that walking helped to improve their mental health, release stress, and promote bonds with their friends or family members. However, different strategies are likely needed to promote community-level cohesion. One such mechanism might be asking participants to involve acquaintances in the technology-based intervention to help bridge the gap between virtual and physical connections.

The lessons learned from this study suggest areas for future research. To achieve the goal of neighborhood cohesion, it may be necessary to redesign the intervention or to build features that are more closely tied to the physical environment. There is clearly a need to try different apps to achieve our goals, most likely those that are compatible with a wearable fitness device. Future studies may focus on the user experience of wearing fitness devices, from which we can examine how social perceptions are derived from a technological intervention as well as any difference between those perceptions in the physical and virtual worlds. Our future research will also aim to investigate in-depth interactions between walking behavior and the corresponding mobile app or devices to discover or modify the interventions that best meet the needs of walking group participants.

## Appendix

Multimedia Appendix 1 Participant characteristics preintervention.

Multimedia Appendix 2 Pre-post survey results: neighborhood cohesion, belonging, physical activity motivation, and self-reported physical activity.

Multimedia Appendix 3 Summary of interview findings.

## Abbreviations

IPAQ: International Physical Activity Questionnaire
MET: metabolic equivalent
PA: physical activity
