# Supplementary material for: A Fitness App for Monitoring Walking Behavior and Perception (Runkeeper): Mixed Methods Pilot Study
Source: JMIR Form Res. 2021 Mar 1;5(3):e22571. doi: 10.2196/22571 (PMC7961398; doi:10.2196/22571)
Supplement: Multimedia Appendix 1 [file formative_v5i3e22571_app1.docx]

Multimedia Appendix 1: Participant characteristics pre-intervention (n=46)

| **Age in yrs, mean (range)** | 45.7 (22 to 70) |
| --- | --- |
| **Years living in neighborhood, mean (SD)** | 13.4 (13.0) |
| **Gender, n (%)** |  |
| Female  Male | 40 (87)  6 (13) |
| **Education, n (%)**  High school graduate  Completed training for trade certificate  or license  Associates Degree  Some years in a 4-year College  College Graduate (Bachelor's Degree)  Post-College Degree (Masters or  Doctorate) | 5 (10.8)  2 (4.4)  4 (8.7)  4 (8.7)  17 (37)  14 (30.4) |
| **Race/ethnicity, n (%)**  White  Asian  Hispanic/Latino | 41 (89.1)  2 (4.4)  3 (6.5) |
| **Relationship status, n (%)**  Single  Married  Divorced/separated  Living with partner | 13 (28.3)  26 (56.5)  4 (8.7)  3 (6.5) |
| **Household living arrangement, n (%)**  Alone  Spouse/significant other only  Spouse and children  Children only  Roommate(s)  Living with parent(s)  Other | 6 (13.3)  13 (29)  15 (33.3)  1 (2.2)  5 (11.1)  3 (6.7)  2 (4.4) |
| **Employment status, n (%)**  Employed full time  Employed part time  Homemaker  Retired  Student  Unemployed  Other | 29 (63.0)  6 (13.0)  1 (2.2)  2 (4.4)  3 (6.5)  2 (4.4)  3 (6.5) |
| **Home ownership, n (%)**  Own  Owned by someone else  Rent | 24 (52.2)  4 (8.7)  18 (39.1) |
