# Supplementary material for: A Fitness App for Monitoring Walking Behavior and Perception (Runkeeper): Mixed Methods Pilot Study
Source: JMIR Form Res. 2021 Mar 1;5(3):e22571. doi: 10.2196/22571 (PMC7961398; doi:10.2196/22571)
Supplement: Multimedia Appendix 2 [file formative_v5i3e22571_app2.docx]

Multimedia Appendix 2

Pre-post survey results: neighborhood cohesion, belonging, physical activity motivation, and self-reported physical activity

| **Outcome** | **Pre (mean, SD)** | **Post (mean, SD)** | **Difference (95% CI)** | **P** |
| --- | --- | --- | --- | --- |
| **Neighborhood Cohesion Index^a^** | | | | |
| Total Score | 2.6 (0.6) | 2.4 (0.6) | -0.15 (-0.25, -0.05) | **.0041** |
| Domain 1 – Attraction to  Neighborhood | 2.2 (0.5) | 2.1 (0.7) | -0.19 (-0.36, -0.01) | **.034^†^** |
| Domain 2 – “Neighboring” | 2.9 (0.9) | 2.7 (0.8) | -0.2 (-0.25, -0.05) | **.012** |
| Domain 3 – Psychological  Sense of Community | 2.5 (0.6) | 2.4 (0.6) | -0.16 (-0.28, -0.04) | **.0076** |
| **General Belongingness Scale^b^** | | | | |
| Total Score | 4.1 (0.6) | 4.3 (0.5) | 0.16 (0.06, 0.26) | **.0031^†^** |
| Domain 1 –  Acceptance/Inclusion | 4.1 (0.6) | 4.3 (0.5) | 0.16 (0.01, 0.30) | **.033^†^** |
| Domain 2 –  Rejection/Exclusion | 4.2 (0.7) | 4.4 (.6) | 0.16 (0.03, 0.29) | **.015^†^** |
| **Physical Activity & Leisure Motivation Scale (PALMS)^c^** | | | |  |
| Total Score | 136.7 (16.1) | 137.1 (133.3) | 0.44 | .83 |
| Mastery | 16.6 (3.7) | 17.2 (3.0) | 0.55 | .22 |
| Physical Condition | 21.1 (2.4) | 21.2 (2.2) | 0.12* | .71 |
| Affiliation | 17.6 (3.8) | 17.6 (3.7) | -0.07* | .898 |
| Psychological Condition | 19.9 (2.5) | 21.0 (2.0) | 1.1 | **.0099** |
| Appearance | 19.0 (3.0) | 18.9 (3.6) | -0.27 | .56 |
| Other Expectation | 13.3 (3.9) | 12.6 (3.0) | -0.67 | .16 |
| Enjoyment | 18.6 (2.5) | 18.9 (2.6) | 0.28 | .41 |
| Competition & Ego | 9.9 (4.0) | 10.0 (3.3) | 0.17 | .66 |
| **IPAQ^d^** | | | | |
| Total MET-min per week | 3726.8 (2600.5) | 3425.9 (2607.1) | -300.9* | .45 |
| Walking MET-min per week | 1513.3 (1119.5) | 1411.1 (1254.7) | -102.2* | .599 |
| Moderate MET-min per week | 1530.0 (1094.7) | 1124.4 (907.7) | -405.6 | **.018^†^** |
| Vigorous MET-min per week | 683.5 (1462.0) | 890.4 (1392.7) | 206.9 | .39 |

^a^1 to 5 scale (1=strongly agree, 5=strongly disagree), so lower scores (closer to 1) indicate greater neighborhood cohesion

^b^ We implemented on 5-point scale (1=strongly disagree, 5=strongly agree). Domain 2 – Rejection/Exclusion questions reverse coded, so higher scores (closer to 5) indicate greater sense of belonging

^c^1 to 5 response scale (1=strongly agree) reverse-coded all responses to match usual scoring scheme. Score obtained by summing. Range for total score 40-200, for each sub-scale 5-25. No pre-determined cut-offs for ‘high’, ‘medium’ or ‘low’ motivation, however midpoint for the total score is 120, and for subscales is 15.

^d^Total activity of 3000 or more MET-minutes/week considered ‘high’, at least 600 MET-minutes/week is ‘moderate’, and anything below ‘low’

^†^Non-significant in sensitivity analysis

^*^Direction change (positive/negative) in sensitivity analysis
