# Supplementary material for: A Fitness App for Monitoring Walking Behavior and Perception (Runkeeper): Mixed Methods Pilot Study
Source: JMIR Form Res. 2021 Mar 1;5(3):e22571. doi: 10.2196/22571 (PMC7961398; doi:10.2196/22571)
Supplement: Multimedia Appendix 3 [file formative_v5i3e22571_app3.docx]

Multimedia Appendix 3: Summary of Findings

| **Theme** | **Subtheme** |
| --- | --- |
| App user satisfaction | Participants were impressed by data tracking which showed the ranking of group members. |
|  | Extra manually operations on the app during walking or running would disturb participants’ walking mood |
|  | If there were not push notifications or push notification not enabled in the app including chat notifications, participants felt the app less meaningful. |
| Satisfaction of walking from this study | Participants would be able to gain motivation for walking after this study. |
|  | If they were organized in groups to walk, participants experience increased feelings of accountability, competitiveness, and peer pressure. |
|  | Participants became more motivated to walk more upon learning their group members’ walk- ranking on the app. |
|  | Some participants benefit from the social networking, which allowed them have chance to meet with others. |
|  | Participants felt that walking was an effect way of releasing stress, especially after their daily work. |
|  | Walking alone made participants feel mindful and at inner peace, which was most meaningful for most participants. |
| Sense of Belonging | To place: Most participants felt a physical connection with their neighborhood. |
|  | To people: Participants felt closer with their friends, families, or the ones they had the most in common with, instead of with their teammates. |
| Walking Patterns (Participants who met criteria, see page 8) | Participants were avid walker, normally most of them walked at least three times a week. |
|  | Participants spent more time walking on their own rather than walking in accordance with the app |
|  | Participants mainly enjoyed walking by themselves. Otherwise, they would rather be accompanied by friends or families with whom they were very familiar |
| Walking Patterns (Participants who didn’t meet criteria) | Participants who didn’t meet criteria walked as frequently as the those who met the criteria. |
|  | Walking frequency and preference of participants in this group were more affected by their various personal life including work and family issues |
|  | Participants in this group were less willing to use the app while walking. |
|  | Participants in this group preferred walking by themselves, no one mention about walking with others. |
